# Editorial: Innovative and sustainable management of organic food and beverage wastes

**DOI:** 10.3389/fnut.2026.1810341

**Published:** 2026-03-05

**Authors:** Gheorghe-Adrian Martău, Francesca Girotto

**Affiliations:** 1 Institute of Life Sciences, University of Agricultural Sciences and Veterinary Medicine, Cluj-Napoca, Romania; 2 Faculty of Food Science and Technology, University of Agricultural Sciences and Veterinary Medicine, Cluj-Napoca, Romania; 3 Department of Environmental Science and Policy, Università degli Studi di Milano, Milan, Italy

**Keywords:** food waste, circular bioeconomy, upcycling, green processing technologies, biocomposites, biomedical biomaterials

The global food and beverage system is characterized by an intrinsic paradox: while striving to meet increasing nutritional demands, it simultaneously generates massive amounts of organic fraction waste that impose significant environmental pressures. Such waste represents not only a substantial economic loss, but a major environmental challenge, contributing an estimated 8–10% of global GHG emissions (Girotto and Beggio). In accordance with the UN SDG 12.3, which targets a 50% reduction in per capita food waste by 2030, there is an urgent need to both strengthen prevention efforts and develop sustainable strategies for managing and valorizing unavoidable food and beverage wastes.

The contributions collected in this Research Topic reflect this dual perspective, spanning prevention-oriented assessments, digital and analytical optimization tools, biological and physicochemical valorization strategies, and advanced material and energy recovery applications.

Recent data reveals the magnitude of the global food waste crisis. According to the 2024 UNEP Food Waste Index Report, approximately 1.05 billion tons of food were generated globally in 2022, of which 60% originated from households (Girotto and Beggio). However, these data capture the post-harvest and consumption stages, excluding substantial upstream losses during agricultural production and processing. The environmental consequences extend beyond the waste itself to encompass all embedded resources, water, energy, and labor required for food production. When such waste fraction is inadequately managed and disposed of in landfills, anaerobic decomposition generates CH_4_, a GHG with a GWP 28 times that of CO_2_ per unit mass over a 100-year horizon (Yip et al.).

The food recovery hierarchy provides a well-established framework for prioritizing food-waste management strategies based on environmental, economic, and social benefits placing prevention and food donation at the top of preferred interventions. Within this framework, the healthcare sector offers a representative example of practical implementation challenges, as food waste from patient services and retail operations can account for up to 20–30% of total hospital waste. Evidence from hospital food services (Yip et al.) shows that the GHG emission reduction potential varies substantially across management options, with donation offering the highest relative benefits but is constrained by the availability of surplus edible food, whereas composting and anaerobic digestion enable the treatment of large and heterogeneous waste streams underscoring the need for integrated strategies that balance environmental performance with operational constraints (Yip et al.).

Artificial intelligence (AI) and digital technologies are emerging as transformative tools for reducing food waste generation and management. While traditional lean management approaches like “*Value Stream Mapping*” have been useful for expert-driven diagnostics, AI extends these capabilities through real-time, scalable, and automated analysis across supply chains (Girotto and Beggio). Digital and analytical innovations operate across multiple, interconnected levels, supporting prevention and redistribution decisions, industrial process optimization, and fine-scale tuning of valorization operations. However, widespread adoption remains hindered by fragmented data infrastructures, elevated costs, and uneven digital readiness. Where data availability or system complexity limits the application of advanced machine learning-based optimization tools, advanced mathematical modeling approaches still play a crucial role in optimizing upcycling processes. For example, Sun et al. demonstrated the application of response surface methodology (RSM) for optimizing microwave-assisted extraction of ursolic acid from apple pomace, supporting the use of green extraction technologies that preserve compound bioactivity.

However, in specific applications, AI-assisted approaches have been shown to enhance process optimization beyond conventional statistical methods. Xiao et al. applied an artificial neural network-genetic algorithm (ANN-GA) framework to optimize extraction parameters for plant protein recovery from safflower seed meal, outperforming RSM. Similar data-driven optimization principles are increasingly being explored across other valorization domains, including biological and energy recovery technologies.

Frontier technologies are expanding the possibilities for energy recovery from the organic waste fraction. Bioelectrochemical and solar-driven reforming technologies are emerging as pathways for energy recovery from organic food waste, enabling the conversion of organic substrates into hydrogen or electricity. Despite their potential for low-carbon energy production, these technologies remain largely at the pre-commercial stage, facing challenges related to scale-up, process stability, and techno-economic feasibility (Girotto and Beggio). Traditional waste-to-energy approaches, including anaerobic digestion, continue to evolve, while insect-based upcycling enables the biological conversion of organic waste into protein- and lipid-rich biomass for feed, biofuels, and high-value compound recovery (Girotto and Beggio).

Lignocellulosic residues from fruit and vegetable processing, including peels, pomace, husks, wheat straw, and bagasse, have strong potential as reinforcing agents in polymer or cementitious matrices, contributing to improved mechanical performance and enhanced end-of-life degradability (1) (Girotto and Beggio). This potential can be further optimized through chemical or physical treatments of natural fibers and the incorporation of nanofillers, which enhance interfacial adhesion with matrix materials and improve tensile strength, impact resistance, and thermal stability. As a result, these biocomposites are increasingly explored for applications in sustainable packaging, automotive interior components, construction materials, and 3D-printed prototypes. However, the absence of unified material standards limits commercial adoption (Girotto and Beggio). Proteinaceous by-products such as feathers, fish scales, and dairy residues represent additional renewable resources, providing keratin, casein, and collagen as functional additives or reinforcing agents.

The conversion of slaughterhouse by-products into biomaterials for regenerative medicine represents a paradigm shift in waste valorization. Corridon et al. proposed a hypothesis-driven strategy for developing sustainable keratoplasty models by repurposing bovine, porcine, or ovine corneal tissues and bladder-derived urine stem cells (USCs) from meat-processing waste. This strategy addresses the critical global shortage of donor corneas, with only one donor cornea available for every 70 patients requiring transplantation worldwide (Corridon et al.).

Despite promising technological advances, several barriers, including ethical concerns, impede the widespread implementation of innovative waste valorization strategies. Fragmented regulatory frameworks, particularly for novel applications like biomedical biomaterials, create uncertainty for scale-up efforts.

Future research should prioritize: (i) standardization of methodologies and performance metrics across valorization technologies and processing approaches; (ii) techno-economic validation of emerging processes at pilot and commercial scales; (iii) development of harmonized regulatory frameworks supporting safe implementation of novel valorization pathways; (iv) systematic integration of LCA and social LCA to quantify environmental and social impacts; and (v) investigation of cascade approaches that anticipate and valorize secondary waste streams generated during primary valorization processes.
